# Inducible Hsp70 in the Regulation of Cancer Cell Survival: Analysis of Chaperone Induction, Expression and Activity

**DOI:** 10.3390/cancers3043921

**Published:** 2011-10-21

**Authors:** Elisa Zorzi, Paolo Bonvini

**Affiliations:** 1 OncoHematology Clinic of Pediatrics, University-Hospital of Padova, 35100 Padova, Italy; E-Mail: elisa.zorzi@libero.it; 2 Fondazione Città della Speranza, 36030 Monte di Malo, Vicenza, Italy

**Keywords:** heat shock protein, Hsp70, cancer, apoptosis

## Abstract

Understanding the mechanisms that control stress is central to realize how cells respond to environmental and physiological insults. All the more important is to reveal how tumour cells withstand their harsher growth conditions and cope with drug-induced apoptosis, since resistance to chemotherapy is the foremost complication when curing cancer. Intensive research on tumour biology over the past number of years has provided significant insights into the molecular events that occur during oncogenesis, and resistance to anti-cancer drugs has been shown to often rely on stress response and expression of inducible heat shock proteins (HSPs). However, with respect to the mechanisms guarding cancer cells against proteotoxic stresses and the modulatory effects that allow their survival, much remains to be defined. Heat shock proteins are molecules responsible for folding newly synthesized polypeptides under physiological conditions and misfolded proteins under stress, but their role in maintaining the transformed phenotype often goes beyond their conventional chaperone activity. Expression of inducible HSPs is known to correlate with limited sensitivity to apoptosis induced by diverse cytotoxic agents and dismal prognosis of several tumour types, however whether cancer cells survive because of the constitutive expression of heat shock proteins or the ability to induce them when adapting to the hostile microenvironment remains to be elucidated. Clear is that tumours appear nowadays more “addicted” to heat shock proteins than previously envisaged, and targeting HSPs represents a powerful approach and a future challenge for sensitizing tumours to therapy. This review will focus on the anti-apoptotic role of heat shock 70kDa protein (Hsp70), and how regulatory factors that control inducible Hsp70 synthesis, expression and activity may be relevant for response to stress and survival of cancer cells.

## 1. Introduction

Growth, proliferation and differentiation of prokaryotic and eukaryotic cells are processes continuously regulated by a strict control of the intracellular protein homeostasis, and deregulation of such processes can have severe consequences on cell survival. The problems of protein folding continuously challenge the cells, and these problems become more critical when cells are subjected to extended levels of stress. Under extreme growth conditions the cells miss the capacity to activate the apoptotic cascade and may undergo necrosis, whereas at lower levels of injury they activate the apoptotic program and undergo a relatively ordered form of death. Necrosis is an unprogrammed, disorderly form of cell death which occurs via rupture of cell walls and organelles, whereas apoptosis is a highly regulated, programmed form of cell death. However, if the level of stress is sufficiently low cells may attempt to survive by producing a set of highly conserved protecting proteins, namely heat shock proteins (HSPs), which assist folding of damaged proteins, preserve them from degradation, and impede toxic intracellular aggregates from threatening cell survival. Heat shock proteins are molecular chaperones that protect cells from extreme physiological, pathological and environmental insults, as well as under normal conditions they are involved in changing protein conformation by promoting multiprotein complex assembly and disassembly. Their rapid induction results in a coordinated series of biochemical and genetic events that are collectively referred as the “heat shock response” (HSR) [1] which involve the maintenance of the steady-state for the majority of the intracellular proteins by either providing stability or driving their proteasomal degradation, the proper folding of denatured or damaged polypeptides, and the inhibition of the apoptotic cascade at multiple levels [2-5]. This explains why proteins adapted to stress like heat shock proteins are so important for the growth and survival of cancer cells, but also why HSPs play critical roles during development and differentiation of untransformed cells [6-10]. A large body of evidence supports the role of the heat shock proteins in maintaining the cancer phenotype, and stressor conditions such those deriving from aneuploidy, copy number variation, and transcriptional alterations have been shown to be responsible for their high expression in tumours. Such a dependence on the normal cellular functions of genes that act in oncogenic pathways in a rate-limiting manner is known as “non-oncogene addiction” (NOA), and heat shock proteins, although not oncogenic *per se*, represent examples of NOA genes for their unusual high expression in cancer and the efficacy of selective therapeutic interventions resulting from their impairment [11-13].

## 2. Structure, Activity and Regulation of Heat Shock Proteins

Mammalian heat shock proteins are generally classified by their relative molecular weight and function into five major families, including the HSP90, HSP70, HSP60, HSP40 and small heat shock proteins family. Members of each family may be expressed both constitutively and induced during stress, and are characterized by chaperone activity controlled, or not, by reaction cycles of ATP binding, hydrolysis and release (Table I). Intracellular localization varies according to function, and heat shock proteins may localize both in the cytosol and nucleus (Hsp27, Hsc70, Hsp70 and Hsp90), in the lumen of the endoplasmic reticulum (Grp78 and Grp94), or reside in the mitochondrial space (Grp75, Hsp60 and Hsp90 homolog TRAP1) [14-16]. In general, HSPs share a molecular architecture consisting of an N-terminal adenine nucleotide binding domain (NBD), a C-terminal domain that includes a peptide/substrate binding domain (PBD/SBD), and a flexible interdomain linker that may both facilitate chaperone ATPase activity (Hsp70) and client recognition (Hsp90) [17-19]. Binding of substrates to the PBD domain occurs through a carboxyl-terminal chaperone motif (EEVD), and is regulated both intramolecularly by NBD nucleotide occupancy, hydrolysis and release, and intermolecularly by the activity of diverse classes of co-factors that interact with both the NBD and the PBD domain in a coordinated sequential manner [20,21]. Co-chaperones and nucleotide exchanging factors (NEFs) regulate substrate affinity and fulfil the ATPase driven cycle of major chaperone proteins, as shown for Hsp70 proteins which bind substrates delivered by co-chaperone J protein Hsp40 and release them upon the interaction with specific NEF co-factors [22,23]. Effective chaperoning activity of Hsp70 in the ‘open’ binding conformation of its PBD/SBD domain is rapid and transient when orchestrated by co-chaperones and NEFs, but iterative if the native state of the client protein is not attained on release [24,25]. J proteins are responsible for substrate binding and Hsp70 ATPase activity, whereas NEF factors catalyze the subsequent ADP release, dictating the duration over which a substrate remains associated to the chaperone. BAG proteins, for example, are nucleotide-exchanging factors that together with Hsp40 help regulate Hsp70 ATPase activity, but also work with Hsp70 in the targeting and selection of certain substrates. BAGs bind the NBD domain of Hsp70 once ATP hydrolysis has occurred and induce nucleotide exchange by stimulating ADP release. The regulatory domain at the C-terminus provides also the site of interaction for tetracopeptide repeat (TPR)-containing proteins, a class of different co-factors that bind and orchestrate nucleotide turnover and substrate binding/affinity using TPR, a 34-aminoacid motif forming an anti-parallel α-helical hairpin, as structural motif. TPR proteins exert multiple functions, and they interact with multiple chaperone proteins to either assist substrates transfer and folding (HOP) or to promote their degradation (CHIP) [26-29].

Co-chaperones generally rise to HSP levels when protein folding is required, and their exact exploitation depends on the type of protein conformational requirements. Raising co-chaperone and NEF levels without a concomitant increase in HSP expression, instead, reduces chaperone activity or may cause a premature release of captured client proteins in the non-native state. In addition, binding specificity of HSPs may be conditioned by structural elements of the non-native protein, such as an increased exposure of hydrophobic amino acids or specific peptide sequences. Hsp70, for example, binds a wide range of unfolded proteins with low sequence selectivity during the early stages of protein folding and misfolding, recognizing extended stretches of hydrophobic nonpolar amino acids in polypeptides undergoing synthesis on ribosomes or in proteins denatured by stress conditions [30,31]. Hsp90, instead, is a heat shock protein that possesses a higher degree of specificity for metastable signalling proteins, including protein kinases, cell cycle regulators and transcription factors, recognizing and binding specific conformational epitopes [32]. Such a diverse substrate specificity depends on the sequence selectivity of the SBD domain, which may recognize unstable polypeptide chains by either the aminoacid composition or the surface features [33-37]. In both cases, molecular chaperones prevent unwanted intermolecular and intramolecular interactions of unfolded proteins, and mask them to premature degradation.

## 3. HSF1 and Modulation of the Heat Shock Response

**Figure 1.**
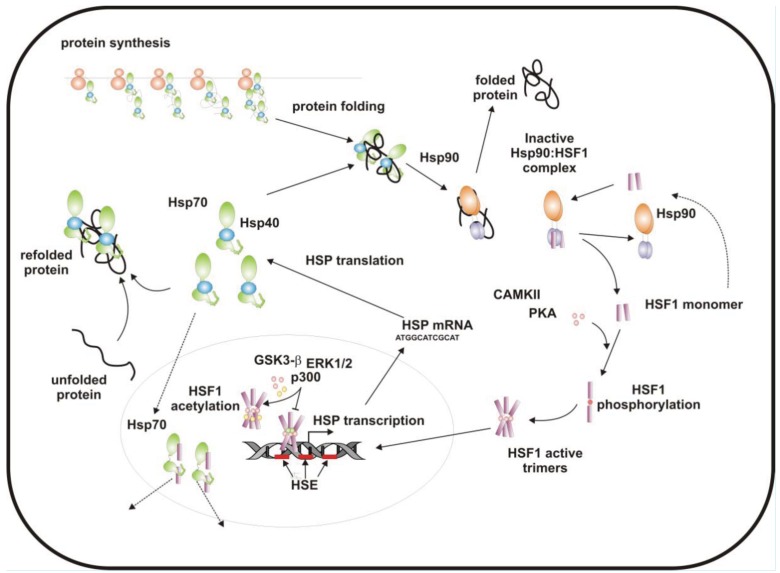
The heat shock protein chaperone system. Heat shock proteins (HSP) are expressed in response to the appearance of unfolded protein substrates. Under non-stressful conditions, HSPs (Hsp70 and Hsp90) bind newly synthesized polypeptides and assist their folding (folded protein), whereas under stressful conditions they recognize denatured, metastable proteins and avoid their unwanted premature degradation (refolded protein). During stress HSPs are induced by HSF1 transcriptional activity, after nuclear relocalization, DNA binding, and phosphorylation (CKII, CamKII) of the protein. Attenuation of HSF1 activity instead involves a negative feedback interaction with both Hsp70 and Hsp90, and additional posttranslational modifications (GSK3b, ERK1/2 and p300-CBP, respectively). HSP: heat shock protein; HSE: heat shock elements.

**Figure 2.**
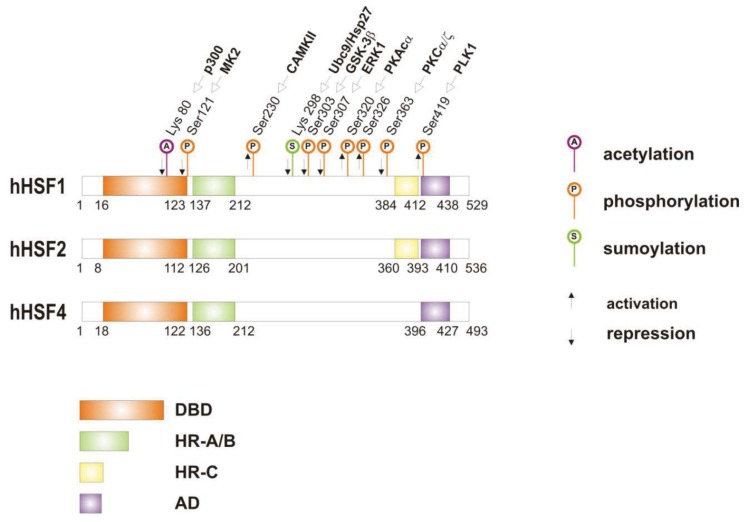
Structural and regulatory features of HSFs. Schematic view of human HSFs structural motifs, corresponding to: DNA binding domain (DBD); hydrophobic heptad repeats (HR-A/B and HR-C); activation domain (AD). The relative position of these domains in HSF1, HSF2 and HSF4 are described by the amino acid residues and by diverse colors. Major HSF1-related post-translational modifications (PTMs) are also indicated, with sites for acetylation (A), phosphorylation (P) and sumoylation (S) distinguished by their contribution to the transactivation function of the protein (activation=↑; attenuation=↓).

When cells are subjected to high temperatures (heat shock) or are exposed to stress, such as oxidative stress, heavy metals or proteotoxic drugs, they respond by increasing heat shock gene expression through the activation of stress-inducible transcription factors (heat shock factors, HSFs). HSFs are transcription factors that bind specific cis-acting sequences upstream of the heat shock gene promoters called heat shock elements (HSE), which consist of multiple adjacent inverted arrays of the 5′-nGAAn-3′ binding site [39-41]. Intermingled to HSE elements there are conventional AT-rich (TATA) and GC-rich (SP1) start sites, CCAAT boxes and AP1/AP2 control elements, suggesting that heat shock genes may be both induced in response to stress and expressed under non-stress conditions [42-44]. TBP (TATA-binding protein) and GAGA are transcription factors that bind HSP gene promoters under physiological conditions, while heat shock factors, like HSF1, regulate HSP expression in response to stressful insults [45-47]. However, binding sites for HSFs may be unmasked by TBP and GAGA proteins as well [48,49], which allow transcriptionally paused RNA polymerase II (RNA Pol II) to remain in place and prime heat shock genes for transcription immediately after stress induction [50-59] (Figure 1). Heat shock factors are transcription factor proteins composed of several functional domains, including a DNA-binding domain (DBD) and two oligomerization domains (HR-A/B) at the protein amino-terminus, a third oligomerization motif (HR-C) and an activation domain (AD) at the carboxyl-terminus, and one or more regulatory domains intermingled to these [58,60,61] (Figure 2). Four major heat shock factors (HSF1-4) have been identified in vertebrates, and three of these, HSF1, HSF2 and HSF4 have been characterized in humans on the base on their function, expression, post-translational modifications (PTMs) and partner protein interactions [38]. HSF1 has a critical role in the regulation of the heat shock stress response and in the expression of HSPs, HSF2 and HSF4 are involved in immunity and differentiation processes, while HSF3 is inactive and maintained in human cells as pseudogene [69,70]. Sequencing of the predicted amino acid regions of HSF1 has provided evidence that exons coding for DBD and HR-A/B domains have been highly conserved among orthologues during evolution, while differences have been accumulating in the HR-C, AD and regulatory domains [68]. The DBD domain consists of helix-turn-helix motifs which form compact globular structures that regulate DNA binding and target-gene recognition [61,63]. The hydrophobic heptad repeat (HR)-A/B domains, instead, mediate both spontaneous and inducible oligomerization of HSF1, by forming a coiled coil conformation typical of leucine zipper-containing proteins [64-67]. HSF1 contributes to many physiological functions during cell growth and differentiation processes, and regulates genes critical for energy production, signal transduction and cytoskeletal organization [71,72]. However, it is much more important for modulating HSP synthesis and stress response, as cells lacking this protein do not develop thermotolerance and are susceptible to stress-induced apoptosis [73,74].

Genetic knockout of HSF1 in transgenic mice, indeed, does not impair organ systems development and birth, but mice lacking HSF1 that are exposed to inflammatory insults or other types of stress have elevated levels of apoptosis [75-77]. Consistently, HSF1 protects cells experiencing constant intrinsic high levels of stress, such as during ageing or tumorigenesis. Both age-dependent collapse of cell proteostasis and tumour-associated increase of protein misfolding are events regulated by HSF1, strengthening the evidence of the unique role of this protein in preventing global instability of proteome in several pathophysiological conditions [78-81].

HSF1 activation occurs as a general response to stress, such as heat shock, oxidative stress and exposure to proteotoxic chemicals, through a series of regulatory events that include dissociation from inhibitor heat shock proteins Hsp70 and Hsp90, HSF1 transition from monomer to trimer, localization to the nucleus, inducible serine phosphorylation, and binding to HSE elements of target gene promoters. Under non-stress conditions HSF1 exists as an inactive monomer in the cytoplasm due to intramolecular bonds between HR-C and HR-A/B domains and its association with Hsp90 and Hsp70 [82-84], whereas in response to stress HSF1 is released from Hsp70 and Hsp90 proteins and forms homotrimers that bind to cis-acting DNA elements HSEs upon phosphorylation [85-91]. Under circumstances of prolonged stress, however, HSF1 is inactivated by negative feedback mechanisms which include the association with newly synthesized HSPs, and additional posttranslational modifications [92-94]. Posttranslational modifications have a critical impact on the HSF1 activation-attenuation cycle, and are finely modulated according to HSF1 subcellular localization and partner-protein interaction [71,95]. Sumoylation is a modification that occurs rapidly and transiently during stress, which is triggered by Hsp27 binding to nuclear HSF1 and HSF1 phosphorylation at Ser303 prior to SUMO (small ubiquitin-related modifier) conjugation at Lys298 [96-98]. Acetylation, by contrast, proceeds more slowly and requires stress-induced p300-CBP (CREB-binding protein) acetyltransferase activity [81,99,100] (Figure 2). While sumoylation impairs HSF1 transactivation activity without affecting DNA binding [101,102], acetylation of the DBD domain attenuates HSF1 binding to HSE elements of stress-responsive genes and inhibits heat shock gene transcription at comparable level. However, when recruited directly at the heat shock gene promoters p300-CBP acetylates histones and participates to HSF1 transcription activity. This occurs in conjunction with STRAP (stress response activator protein), a DNA damage-responsive protein that regulates chromatin-bound levels of both phosphorylated HSF1 and active p300-CBP. Cells depleted of STRAP, indeed, do not activate HSF1-dependent transcription of heat shock proteins, and have impaired survival under stressful conditions [103-107]. As regard to phosphorylation, 12 serine residues participate in the regulation of HSF1 transcriptional activity [108], and among these Ser230, Ser320, Ser326 and Ser419 activate HSF1, whereas Ser121, 303, 307 and 363 contribute to its repression [88,109,110]. Deletion mapping analysis has demonstrated that Ser307 and Ser303 are constitutively phosphorylated in a sequential manner by GSK3B and ERK1/2 MAP kinases, respectively, and inhibit HSF1 transcriptional activity during stress recovery, whereas serine 363 undergoes phosphorylation upon PKCα and ζ expression [109] and inhibits HSF1 transactivation activity rather than HSF1 binding to HSE elements [111].

By comparison, HSF1 is activated by phosphorylation at Ser230 and Ser326, which stimulates the transcriptional activity of DNA-bound HSF1, and by phosphorylation at Ser419 and Ser320 that modulates HSF1 oligomerization, nuclear translocation and DNA binding, respectively [88,108]. Protein kinases that regulate Ser230, Ser419, and Ser320 phosphorylation are calcium/calmodulin-dependent protein kinase II (CAMKII), PLK1 (polo-like kinase 1), and protein kinase A (PKAcα), respectively, whereas protein kinases responsible for phosphorylation of HSF1 at Ser326 are not known [88,112]. In this context, modulation of HSF1 phosphorylation may be of therapeutic benefit in the treatment of diseases that have an underlying abnormality in protein conformation and activity, as demonstrated in cancer cells where HSPs help to maintain the malignant phenotype, and their levels may further induced by chemotherapy as side effect of treatment. This has been demonstrated for several stress-related kinases, including p38MAP kinase, GSK3β and JNK [113-118]. Consistently, proteasome inhibitors, a well-known class of potent anti-cancer agents, up-regulate HSPs synthesis in cancer cells by stimulating hyperphosphorylation and DNA binding of HSF1, and this occurs through enhanced expression and activity of p38MAPK kinase [119-121]. Likewise, exposure to heat shock and oxidative stress activate p38MAPK and enhance HSF1 hyperphosphorylation, while p38MAPK inhibition reduces the rise of Hsp70 levels brought about by these harmful conditions and enhances stress-induced apoptosis (). Treatment with compounds that activate GSK3β, JNK and ERK1/2 MAP kinases may be equally effective at inhibiting Hsp70 expression and chaperone-mediated cytoprotection in transformed cells, as these protein kinases affect negatively HSF1 transcriptional activity and heat shock gene expression, as previously demonstrated [122-125].

## 4. Regulation and Expression of Inducible Hsp70 Chaperone Protein

**Figure 3.**
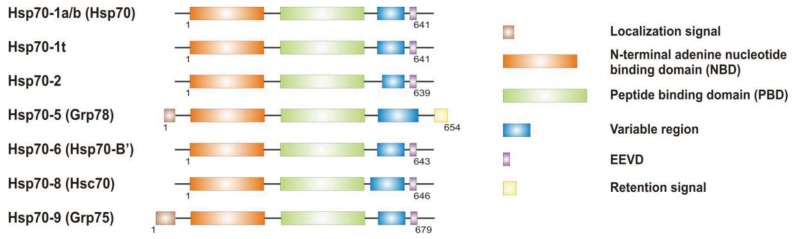
Structure and domains of human Hsp70 family member proteins. Human Hsp70s contain distinct functional domains, including a 30 kDa peptide binding domain (PBD), responsible for substrate binding; a 45 kDa nucleotide binding domain (NBD), which includes the ATP-binding cleft domain; and two other domains shared by most of the family members: a carboxyl-terminal EEVD motif, or chaperone motif, and a variable region at the EEVD N-terminal. More specific localization and the retention signals are found in Hsp70-5 and Hsp70-9 proteins, to guide chaperone localization into the endoplasmic reticulum (ER) or the mitochondrial lumen, respectively.

It is now clear that cancer arises through a multistep, mutagenic process whereby transformed cells acquire a common set of properties including unlimited proliferation potential, self-sufficiency on growth signals, and resistance to antiproliferative and apoptotic cues. Many of these phenotypic traits can be brought about by numerous genetic alterations, which alongside gain-of-function mutation and amplification of oncogenes, or loss of function of tumour suppressor proteins, include overexpression of key proteins with non-oncogenic functions. Besides, cancer cells have extensively rewired pathways for growth and survival that underline their malignant phenotype, and thus it appears important to identify either critical nodes or functional regulator proteins in the oncogenic network whose inhibition may result in system failure, that is apoptosis. As previously mentioned, tumours exhibit proteotoxic stress evidenced by their frequent constitutive activation of the heat shock response, and heat shock proteins are key players of many stress support pathways that help cancer cells to cope with the stress of their oncogenic state [126]. Among them, Hsp70 proteins are critical for growth and survival of transformed cells, because they serve both as chaperones, assisting folding of nascent polypeptides and the intracellular localization of native proteins, as they act to protect cells to repeat exposure of noxious stimuli which would otherwise cause lethal molecular damage and induce apoptosis. Eukaryotic cells possess several structurally similar Hsp70 family member proteins, but the fact that their simultaneous deletion is lethal for the cells suggests that Hsp70 proteins may serve several overlapping chaperone functions but also perform specific activities that cannot be compensated by others. In most of the cell types heat shock 70kDa proteins are detectable at significant levels in unstressed cells and further increased in abundance on stress [127,128]. Whereas inducible protein expression is regulated by HSF1 during stress, in unstressed conditions transcription factors different from HSF1 (*i.e.*, STAT, CCAAT-box and SP1) modulate basal protein expression [129]. Eight Hsp70 family member proteins have been identified, and distinguished on the base of their amino acid composition, expression rate, subcellular localization and stress responsiveness. Among them, Hsp70 (Hsp70-1a; Hsp70-1b) and Hsp70-6 (Hsp70-B′) are stress-inducible family member proteins, whereas Hsp70-1t, Hsp70-2, Hsp70-5 (Bip, Grp78), Hsp70-8 (Hsc70) and Hsp70-9 (Grp75, mortalin) are chaperone protein expressed constitutively in a tissue specific-manner [126] (Figure 3).

Under normal growth conditions Hsp70s function as ATP-molecular chaperones and assist protein folding. On stress Hsp70 proteins cope with the increased concentration of unfolded and denatured proteins, and prevent toxic aggregates from threatening cell survival by inducing apoptosis [130,131]. Such an ability to disable apoptosis relies on the capacity of Hsp70s to interact with multiple unstable components of the programmed death machinery, including pro- and anti-apoptotic proteins, DNA-damaging factors, or stress protein kinases. Most of the studies published on these proteins deal with the two major members of the Hsp70 family, the inducible Hsp70 molecular chaperone (also known as Hsp72) and cognate protein Hsc70. Inducible Hsp70 is critical for transformation, proliferation, poor differentiation and invasion of cancer cells, whereas Hsc70 provides general assistance to normal cells [132-134]. Unlike Hsc70, which is constitutively expressed in non-tumor cells and tissues, Hsp70 is present at relatively low levels in untransformed cells and it's rapidly induced upon stress. Depletion of inducible Hsp70 however does not affect cell survival but results critical to withstand stressful insults induced by UV exposure, osmosis, and hyperthermia [135-138]. Conversely, constitutive high levels of Hsp70 are frequently observed in cancer cells, in which Hsp70 confers resistance to stress-induced apoptosis, serves in suppression of default senescence, and is associated with metastasis development and drug resistance [139-141]. In tumors Hsp70 may be also expressed regardless HSF1 transcriptional activity, consistent with the notion that different factors other than HSF1 may regulate Hsp70 status and activity [142]. STAT1, STAT3 (signal transducer and activator of transcription) and NF-IL6/CEBPB are the transcription factors responsible for Hsp70 synthesis in the absence of stress [143,144], as inhibition of both STAT and NF-IL6 activity reduces basal expression of Hsp70 chaperone protein in cancer cells lacking HSF1 or expressing a defective protein [142,145-147]. Whereas STAT3 binds sequences at the *hsp70* promoter (5′-CTGGRA-3′) different from HSE elements and competes with HSF1 for Hsp70 expression on stress [148-150], STAT1 induces Hsp70 expression by recognizing different elements in the promoter region (GAS, interferon Gamma Activated Sequences), and does not compete with HSF1 for transcription [151]. By comparison, NF-IL6 (nuclear factors IL6) proteins (*i.e.*, CCAT/enhancer-binding protein, C/EBP) recognize STAT-responsive elements and cooperate with HSF1 for inducing Hsp70 [152], but they can also bind Hsp70 proximal promoter regions together with STATs and produce stronger activation of Hsp70 transcription in the absence of stress [153,154].

## 5. Anti-Apoptotic Activity of Hsp70

**Figure 4.**
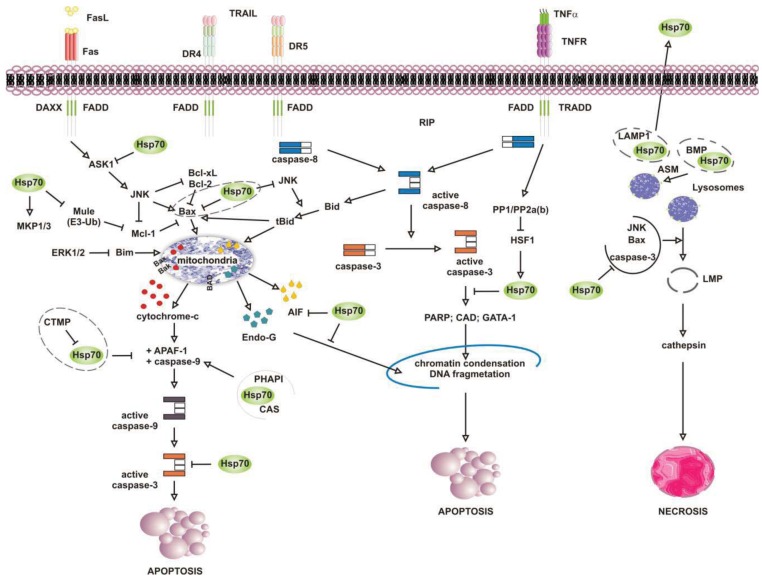
An anti-apoptotic role of human Hsp70. Cytoplasmic Hsp70 is a decisive negative regulator of both the extrinsic and intrinsic apoptotic pathways. At a pre-mitochondrial stage, Hsp70 inhibits receptor-mediated activation (DR4/5; TNFR; TRAIL; Fas) of stress kinases (SEK; JNK) or kinases directly involved in the induction of apoptosis (ASK1), as well it impairs the activation of initiator caspases (caspase-8 and -9), effector caspases (caspase-3) and pro-apoptotic molecules (Bid, Bim and Bax). At a mitochondrial level, Hsp70 prevents mitochondrial membrane permeabilization, and antagonizes cytochrome c and Endo-G release through inhibition of Bax activity. Downstream to mitochondria, Hsp70 protects cells from apoptosis by interacting with and inhibiting AIF and Apaf-1 pro-apoptotic activity, thereby impairing apoptosis execution. Hsp70 can also stabilize lysosomes and lysosomal membrane permeability (LMP), preventing the release of acidic hydrolases (cathepsins) from the lysosomal lumen.

Numerous studies have attributed the survival function of Hsp70 to the ability to suppress the engagement of apoptosis, since cells that contain elevated levels of this chaperone have an increased ability to tolerate different types of stress at several point of the apoptotic cascade [155-157] (Figure 4). Stress-inducible Hsp70, in fact, acts as a natural inhibitor of several stress-kinases at the beginning of cell death, as well as it protects cells from apoptosis by binding and modulating the activity of various pro- and anti-apoptotic proteins at transcriptional and posttranslational level. By impairing Janus kinase (JNK) activity, in fact, Hsp70 prevents JNK-induced phosphorylation and inhibition of Bcl-2 and Bcl-×L anti-apoptotic proteins, and promotes cell survival through the maintenance of mitochondria stability. Similarly, Hsp70 when induced prevents Bid-mediated damage to mitochondria and inhibits the release of pro-apoptotic cytochrome c and SMAC from these organelles through the inhibition of SEK1-dependent phosphorylation of JNK and JNK-mediated activation of Bid [158-160]. Hsp70, thus, can prevent stress-induced activation of JNK maintaining the levels of inactive dephosphorylated JNK or, alternatively, inhibiting its activating phosphorylation by SEK1. Either scenario, however, leads to a block in JNK signaling and helps to prevent stress-induced JNK-mediated loss of survival. Conversely, JNK counteracts Hsp70 anti-apoptotic activity and facilitates cell death by phosphorylating HSF1 and inhibiting the transcription of Hsp70 [160-163]. In a similar fashion, Hsp70 modulates ERK1/2 MAP kinase activity during stress, through the activation of MKP1 and MKP3 phosphatases [164]. Tumor cells that overexpress Hsp70 are more resistant to stress because of MKP-dependent dephosphorylation and inactivation of pro-apoptotic ERK1/2, and do not undergo stress-induced cell death [165-168]. In contrast, the accumulation of phospho-ERK1/2 is typical of Hsp70 knock-out cells undergoing apoptosis that do not exhibit MKP1 and MKP3 activity. Likewise, Hsp70 competes with RAF for binding co-chaperone Bag-1, which supports RAF-induced activation of ERK1/2. Through the association with Bag-1, Hsp70 prevents phosphorylation of ERK1/2 by RAF and helps to maintain survival of cancer cells exposed to stress [169-170].

Release of pro-apoptotic factors from mitochondria is a second pivotal point within the intrinsic apoptotic pathway which is regulated by Hsp70, since Hsp70 binds and alter expression and activity of several pro- and anti-apoptotic molecules, including Bcl-2 family member proteins Bax and Bak [171-174]. Exposure to heat has been shown to alter multidomain pro-apoptotic Bax protein, which oligomerizes and inserts into mitochondria, creating transition pores at the outer membrane that serve for the release of apoptogenic factors cytochrome-c, SMAC/Diablo and AIF at the beginning of the apoptoic cascade [175-177]. Under these circumstances, JNK-dependent phosphorylation and inhibition of 14-3-3 proteins renders Bax free and competent for mitochondrial insertion, unless recognized and maintained inert in the cytoplasm by Hsp70 [178,179]. At the same time, JNK phosphorylates and primes anti-apoptotic Mcl-1 protein for degradation via the interaction with Mule ubiquitin ligase, preventing Mcl-1 from binding and inhibiting activated Bax. By binding JNK and Mule, Hsp70 prevents Mcl-1 degradation, which in turn binds and impedes Bax from forming transition pores at the mitochondrial membrane [180]. Indeed, chronic myeloid leukemia (CML) cells that express BCR-ABL oncogenic kinase exhibit increased levels of hyperphosphorylated HSF1, inducible Hsp70 and anti-apoptotic Mcl-1 proteins, while cells in which BCR-ABL is inhibited have less Hsp70, and the activation of pro-apoptotic Bax induced by imatinib leads to cell death [181]. Patients who relapse to imatinib have an even larger increase in Hsp70 and Mcl-1 levels, and drug resistance is largely ascribed to the anti-apoptotic activity of these two proteins [182-188].

Downstream to mitochondria Hsp70 possesses the ability to inhibit the formation of functionally competent apoptosome by directly associating to Apaf-1 (apoptotic protease activating factor 1) CARD domain (caspase recruitment domain) and masking it to initiator caspase 9 [189,190]. The ATPase activity of Hsp70 is crucial for binding Apaf-1, since complex formation does not occur in the absence of ATP or in the presence of an ATPase dead Hsp70 protein [191]. However, survival activity of Hsp70 may be counteracted by proteins located in the cytoplasm or released from mitochondria on stress, such as carboxyl-terminal modulator CTMP protein, CAS and PHAPI. CTMP localizes at the plasma membrane, where it acts as tumor suppressor protein of oncogenic PKB/Akt kinase, or resides in the intermembrane mitochondrial space, whereas CAS and PHAPI localize mainly in the cytoplasm. Upon phosphorylation CMTP is released from mitochndria and binds Hsp70 prior to its association with both Apaf-1 and cytochrome c [192]; cellular apoptosis susceptibility protein CAS and tumor suppressor PHAPI, instead, associate and prevent Hsp70 protein from binding Apaf-1 in conditions of low-expression Hsp70 levels, since on stress inducible Hsp70 binds Apaf-1 and cytochrome c despite CAS and PHAPI expression [193]. Hsp70 overexpression provides also resistance to Apaf-1-independent apoptosis, like that promoted by AIF (apoptosis inducible factor). AIF plays a role in survival of several tumour cell types, and like cytochrome c it effluxes from mitochondria in response to damages induced by noxious stimuli. Cytoplasmic AIF translocates into the nucleus where it induces caspase-independent chromatin condensation and DNA fragmentation [194-196] In the presence of Hsp70 nuclear import and death-inducing activity of AIF are prevented, whereas silencing of Hsp70 or inhibition of its ATPase activity enhance AIF pro-apoptotic functions and stimulate cells killing [191,197,198]. Notably, AIF mutants that lack the N-terminal portion of the protein are not inhibited by Hsp70, suggesting that this region is recognized by Hsp70 when AIF effluxes from mitochondria [199,200]. Mapping more in detail the AIF binding domain has permitted to design AIF-derived decoys for Hsp70, capable of sensitizing transformed cells to drug-induced apoptosis by competing with endogenous AIF for binding Hsp70 [201]. Similarly, Hsp70 associates and inhibits endonuclease G (EndoG), a DNAse responsible for caspase-independent disruption of nuclear integrity [202], although since EndoG forms a complex with AIF, the Hsp70/EndoG association might be an indirect consequence of Hsp70 binding to AIF [203].

At the plasma membrane the scenario is further complicated by what is known as the “heat shock paradox”, the seemingly ability of Hsp70 to induce both cytoprotection and cytotoxicity in response to different exogenous stimuli that activate TRAIL-R, TNFR1 and FasR death receptors. Death receptors are protein tyrosine kinases that oligomerize upon ligand binding and recruit a multitude of downstream proteins in what is known as “death-inducing signaling complex” (DISC). Proteins at DISC that bind death receptors directly or through specific adaptor proteins (*i.e.*, FADD and TRADD) engage multiple intracellular signals that promote apoptosis through activation of either caspase 8 or the ASK-1/JNK pathway. With respect to this, Hsp70 has been reported to interact with DR4 and DR5 TRAIL receptors and affect ligand-induced assembly of DISC complex in both normal and tumor cells [204,205]. However, unlike normal cells that appear to be refractory to death receptor-induced apoptosis, tumor cells are sensitive to TRAIL receptors activity and undergo apoptosis if not protected by Hsp70 [206-208]. This occurs in cells that require amplification of the apoptotic signals through mitochondria injury (type II), whereas not in the cells that activate the apoptotic cascade without mitochondrial involvement (type II cells) [209]. This is consistent with the findings that Hsp70 does not bind and modulate initiator caspases at the plasma membrane (*i.e.*, caspase-8) but it functions downstream to mitochondria where it prevents the activity of effector caspase 3. In a similar fashion, Hsp70 inhibits apoptosis induced by TNFα and Fas ligands, through the inhibition of receptor-induced JNK-mediated mitochondrial injury and caspase 9 activity [210-212]. Under these conditions Hsp70 prevents cell death induced by TNFR1 receptor through the inhibition of ASK-1 (apoptosis signal-regulating kinase 1) and JNK kinase activity [163,213]. This confers protection from JNK-induced phosphorylation of BH-3 pro-apoptotic protein Bid and Bid-mediated mitochondrial damage, giving long term survival advantage to cancer cells [214,215]. TNFR1, however, may counteract Hsp70 anti-apoptotic activity under the same circumstances, as the activation of PP1/PP2a and PP2b phosphatases by TNFR1 can modulate phosphorylation and activation of HSF1 and thereby HSF1-dependent Hsp70 transcription [216]. Under some circumstances, however, apoptosis may be enhanced by Hsp70 rather than being prevented, as shown when Hsp70 binds and stimulates mutant p53 to bind TRAIL-R1 and -R2 gene promoter regions, or when Hsp70 accumulates at the plasma membrane of tumour cells [208,217]. In the first case, pro-apoptotic activity of Hsp70 results in p53-mediated enhanced transcription and expression of TRAIL receptor proteins, whereas with respect to Hsp70 localization at the plasma membrane the underlying mechanism appears to involve an enhanced uptake of death-inducing granzyme B protein through its association with Hsp70. Similarly, Hsp70 may promote TNF-dependent FADD-mediated activation of caspase-8, by limiting anti-apoptotic proteins FLIP (FLICE inhibitory protein) and RIP1 (receptor-interacting protein 1) recruitment at the plasma membrane, or by inhibiting IKK-mediated phosphorylation and proteasomal degradation of NF-kB inhibitor IkB and, thus, NF-κB pro-survival signaling [218-221].

## 6. Role of Inducible Hsp70 Chaperone in Lysosome-Mediated Cell Death

It is well established that lysosomes play an active role in the execution of both apoptosis and necrosis, and a range of stress stimuli coming from outside and inside the cells lead to lysosomal membrane permeabilization (LMP) [222-225]. The primary function of lysosomes is degradation of proteins, and for such a purpose these organelles are filled with more than 50 different types of acid proteases [226,227]. Trafficking and stability of lysosomes is important for tumour growth and progression, since lysosomes rupture and intracellular release of their content is lethal, while the pericellular and extracellular release of it may facilitate matrix disruption, angiogenesis and tumour invasion [228,229]. In this context, Hsp70 has been shown to accumulate at the lysosomes of many tumour cell types, acting as safeguard of membrane integrity [230,231]. By interacting with LMP-inducers Bax, JNK and p53, as previously mentioned, Hsp70 may prevent lysosomal membrane permeabilization and proteolytic hydrolases release from damaged organelles, preserving cells from self-distruction [232,233]. Accordingly, Hsp70 downregulation in cancer cells causes both intracellular Ca^2+^ increase and loss of lysosomes integrity, resulting in massive cell death by LMP [234,235] (Figure 4). This has been shown in glioblastoma, breast and colon carcinoma cells, in which the inhibition of Hsp70 leads to both cathepsin-mediated lysosomes leakage and stress-induced cell death [236,237]. Neuronal apoptosis and necrosis following cerebral ischemia also occur via Hsp70-dependent regulation of lysosomes stability, like the upregulation of Hsp70 is effective at reducing cerebral infarction after oxidative ischemia through protection of lysosomal integrity [231].

At a molecular level Hsp70 is shuttled to late endosomal/lysosomal membranes by endo-lysosomal anionic phospholipid bis(monoacylglicerol)phosphate BMP protein, an essential co-factor for sphingomyelin metabolism [238-240]. Herein, Hsp70 sustains the activity of acid sphingomyelinase protein ASM, which controls phospholipids concentration and steric conformation of lysosomal membranes, as well as trafficking of these organelles across the cell [241]. Consistent with these findings, Hsp70, BMP and ASM are frequently overexpressed in tumour cells resistant against stress-induced lysosomal damages, whereas Hsp70 expression and activity is significantly reduced in patients affected by severe lysosomal storage disorders. Furthermore, whereas cognate Hsc70 chaperone protein localizes at lysosomes bound to lysosome-associated membrane protein 2a (LAMP2a) and permits misfolded proteins to be imported and degraded, inducible Hsp70 chaperone may also interact with LAMP1 protein, and be transported by this at the plasma membrane to be released outside during inflammation and immunity processes [222,242-244]. Accordingly, heat shocked cell lysates have enhanced vaccine activity compared to non-heat shocked controls, and forced expression of Hsp70 in tumours has been demonstrated to induce a powerful T cell mediated immune response [245,246]. When expressed at the surface of tumour-derived exosomes (TDE), indeed, Hsp70 may promote myeloid-derived suppressor cells (MDSCs) activity and restrain tumour immune surveillance of CD8^+^ cells [247,248]. TDE-associated Hsp70 binds Toll-like receptor 2 (TLR2) on MDSC plasma membrane and triggers MSDC immunosuppressive function through IL-6 production and STAT3 activation [249]. Tumour growth and drug resistance are both increased in TDE-presenting Hsp70-expressing cells, whereas exosomes depletion by efflux pump inhibitors reduces extracellular Hsp70 levels and enhances chemotherapy efficacy [250].

## 7. Targeting Hsp70

Considering the possible therapeutic potential of modulating Hsp70 activity in tumours, extensive work in recent years has focused on the identification of Hsp70 inhibitors to use alone or in combination with existing anti-neoplastic drugs. Agents which modulate Hsp70 expression and activity may be of particular benefit for the treatment of cancer, consistent with the notion that transformed cells are extremely dependent on heat shock proteins to maintain their constitutive state of stress, and several anti-cancer agents, including classical DNA intercalating molecules and more selective targeted therapeutics, induce HSP expression as a side effect of treatment. In this context, inhibitors of HSF1 and direct modulators of Hsp70 activity have been successfully tested as anti-cancer drugs, offering therapeutic benefits and selective effects on transformed versus untransformed cells [158,230,251-253]. With respect to this, the naturally occurring flavonoid quercetin (3,3′,4′,5,7-pentahydroxyflavone) is one of the first Hsp70 inhibitors that was shown to reduce the expression of Hsp70 by the inhibition of HSF1 phosphorylation and transcriptional activity, and to sensitize tumour cells to apoptosis induced by anti-cancer drugs by inhibiting Hsp70 and Mcl-1 expression, while activating pro-apoptic Bax [254-257]. However, quercetin effectiveness was found to be dependent on the concomitant expression of several functional proteins, including the multidrug resistance protein MRP [258-260]. HSF1, in fact, plays a role in P-glycoprotein (P-gp) expression and activity, and incubation with quercetin prior to administration of anti-cancer drugs appears to be more effective at inhibiting MRP-mediated drug efflux and to enhance drugs‧ effectiveness [261,262]. In addition, quercetin inhibits expression and activity of protein kinases involved in growth and survival of cancer cells, including few critical for HSF1 transcriptional activity and Hsp70 expression [263]. Casein kinase 2 (CK2) and calcium/calmodulin kinase II (CamKII) are among these proteins, as their inhibition by quercetin prevents activating phosphorylation of HSF1 at Thr142 and Ser230, respectively. Good inhibition of Hsp70 induction correlates with good inhibition of both CKII and CamKII kinases, whereas quercetin derivatives that show poor inhibition of either or both proteins have little or no ability to inhibit stress-induced Hsp70 expression [88,264]. Besides, quercetin may also act by inhibiting Hsp70 expression post-transcriptionally without direct effect on HSF1 activity. It has been shown that exposure of HeLa cells to quercetin favours AMP-activated protein kinase (AMPK) activity and reduces Hsp70 levels [265,266]. While in the absence of quercetin AMPK is inhibited by phosphatase 2A (PP2A) protein and Hsp70 is expressed, in the presence of quercetin, activated AMPK reduces Hsp70 mRNA stability and sensitizes cancer cells to stress-induced apoptosis [267]. Parallel to inhibition of Hsp70 expression, however, quercetin may cause phosphorylation of Hsp27 (Ser78) in the absence of heat shock, which counteracts the lethal effects achieved by Hsp70 depletion [266]. Quercetin derivatives that enhance phosphorylation of Hsp27 are indeed not as good stress inhibitors as derivatives capable of inhibiting both CK2 and CamKII without stimulating Hsp27 activity, since phosphorylation is a pre-requisite for Hsp27 anti-apoptotic activity [266].

Hsp70 can also be modulated more directly, as observed in cells treated with *N*-formyl-3,4-methylenedioxy-benzylidene-γ-butyrolaetam (KNK437) and diterpene triepoxide triptolide inhibitors. Interestingly, KNK437 alters stress-inducible Hsp70 binding to Hsp40, whereas triptolide inhibits the expression of Hsp70 by interfering with the activity of HSF1 C-terminal transactivation domain [268-270]. However, in combination with cytotoxic agents such as proteasome inhibitors and modulators of Hsp90 ATPase activity (*i.e*., 17-allylaminodemethoxygeldanamycin) both KNK437 and triptolide enhance drug-induced apoptosis and increase the loss of clonogenic survival of transformed cells, by downregulating Hsp70 expression and activity prior to the drug treatment [271-280]. This has been shown also by using two other inhibitors, NZ28 and emunin, which prevent Hsp70 expression and activity in both heat shocked cells and in cells treated with either proteasome or Hsp90 inhibitors [281-283]. These inhibitors are relatively specific compared to those described above, as both emunin and NZ28 inhibit Hsp70 translation rather than HSF1 activity, precluding Hsp70 expression only.

Blocking HSF1-dependent pTEFb recruitment at *Hsp70* promoter may be an even more interesting approach to impede inducible Hsp70 expression, using decoys that interfere with the HSF1/cdk9 complex formation. Since HSF1 is required for p-TEFb protein (positive transcription elongation factor b) to be placed at the stress-responsive gene promoters primed with paused RNA polymerase II, molecules that associate with HSF1 and inhibit pTEFb recruitment may reduce Hsp70 transcription, thereby compromising cell survival [284,285]. Based on these findings, the possibility of modulating the Hsp70 ATPase activity may have even more promising therapeutic potential than the inhibition of chaperone expression. This has been shown with 15-deoxyspergualin (15-DSG) derivatives and different adenosine-derived inhibitors, which bind the C-terminal EEVD motif and the ATPase domain of Hsp70, respectively, and affect both the endogenous and Hsp40-stimulated ATPase cycle of Hsp70.

One interesting aspect of Hsp70 biology that remain to be more fully elucidated is the ability of Hsp70 to form multiprotein complexes with a wide range of molecules, including co-chaperones partner proteins and signal transducers. Targeting the interaction between Hsp70 and its regulatory partners would be expected to control specific chaperone activities, and have more limited toxicity because of reduced global impairment of protein homeostasis. In support of this idea, peptide aptamers that inhibit the anti-apoptotic function of Hsp70 by acting as substrate mimetic peptides have been recently developed, like ADD70, a construct encoding the minimum binding region of the Hsp70 substrate AIF. ADD70 when used alone is not cytotoxic *per se*, because it does not bind to Hsc70 or any other heat shock protein with housekeeping function. However when transduced in tumour cells ADD70 promotes stress-induced apoptosis by its unique capacity to interact with and sequester inducible Hsp70 chaperone protein from binding AIF [201,286]. Similar to this, a small-molecule Hsp70 inhibitor called 2-phenylethylenesulfonamide (PES) has been identified, and shown to alter the activity of Hsp70 in multiple cellular processes. PES interacts with the C-terminal SBD domain of Hsp70 and plays a critical role in determining Hsp70 partner selectivity. In the presence of PES, Hsp70 does not bind Hsp90, Hsp40, BAG and CHIP chaperones and co-chaperones anymore, and the normally cytoprotective role of Hsp70 is disabled. In this regard, cancer cells that overexpress Hsp70 and experience high levels of metabolic stresses are extremely sensitive to PES administration, and undergo readily cell death through caspase-independent mechanisms that involve increased protein aggregation, impairment of lysosomal functions, and inhibited autophagy.

## 8. Concluding Remarks

Nowadays, great clinical interest is focused on the design of novel therapeutic strategies that simultaneously inhibit multiple signaling pathways in cancer cells, since drug resistance frequently occurs in cells that are characterized by high genomic instability and the accumulation of somatic mutations. At any given time, a living cell is a multi-tasking system in which a plethora of external and internal stimuli continuously challenges its survival. Tumor cells are particularly threatened by harmful growth conditions, and a specific and rapid response to stress becomes necessary to maintain transmission of signals across different pathways that protect cells from death. Multiple perturbations of homeostasis can have severe consequences for the cell, and an excessive cell death may be very costly for the entire organism as well. In such a context, multiple checks need to be guaranteed by the cell to survive, and heat shock proteins, by the nature of their functions, provide such critical links between signaling networks and molecules. Contrary to their generic name HSPs do not just reverse stress-induced protein misfolding or help nascent polypeptides to fold properly, but have a more global role in cell metabolism, including protection from cell death. While stress-inducible heat shock proteins are barely detectable in normal cells, their expression is enhanced in many tumors, where they favor adaptation to adverse microenviromental conditions and contribute to the development of resistance to a wide range of pharmacological agents. Therefore, heat shock proteins may be considered potential drug targets, given their widespread roles in both nonstress and stressful conditions. Hsp70 has gathered significant attention as a potential drug target over the past few years, and the importance of its modulatory function has been demonstrated in a wide range of pathophysiological states, including cancer and neurodegenerative diseases. Hsp70, in fact, broadly shapes cell homeostasis, by affecting protein quality control and turnover, and when this occurs upon stress induction, as observed constantly in transformed cells, it provides survival advantages by binding and inhibiting multiple components of the caspase-dependent and –independent apoptotic pathways. Hsp70 plays roles also in lysosomes integrity, consistent with its known substrate promiscuity and pleiotrophic activity. Such a seemingly ubiquitous nature of the anti-apoptotic effects of Hsp70 may be interpreted as a lack of specificity and biological relevance, but it's now clear that in this way Hsp70 provides major support to tumorigenicity and resistance of cancer cells to noxious insults. The fact that the cytotoxic effects of Hsp70 down-modulation are so important for cancer cells survival is consistent with the notion that tumors are particularly dependent on HSP expression and activity, and that their constitutive stressed phenotype requires the continuous cytoprotective action of molecular chaperones like Hsp70. As a consequence, extensive efforts from many laboratories have provided insights of the complex regulatory mechanisms that control Hsp70 expression and activity, as well as novel cancer therapeutics that target Hsp70 activity have been developed. However, many questions remain before Hsp70 can be considered a fully validated drug target, and how to practically manipulate Hsp70 activity to have more limited toxicity remains a mandatory challenge that has to be addressed to make Hsp70 a good therapeutic target for clinical intervention.

## Figures and Tables

**Table 1.** Nomenclature, location and function of the major heat shock protein families.

**Family**	**Organism**	**Chaperones**	**Location**		**Function**
**Hsp100**	E.coli	ClpA, B, C	cytosol	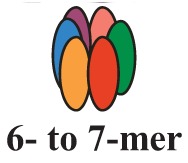	Role in stress tolerance; it helps the resolubilization of heat-inactivated proteins from insoluble aggregates
	S.cervisiae	Hsp104	cytosol		
**Hsp90**	E.coli	HtpG	cytosol	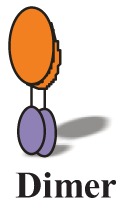	Role in signal transduction (e.g., interaction with steroid hormone receptors, tyrosine kinases, serine/threonine kinases); regulation of heat shock response; role in cell cycle and proliferation; maintenance of mitochondrial integrity
	S.cervisiae	Hsp83	cytosol		
	Humans	Hsp90	cytosol,		
		GRP94	nucleus		
		TRAP1	ER		
			mitochondria		
**Hsc/Hsp70**	E.coli	DnaK	cytosol	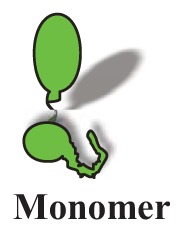	Roles in lambda phage replication; regulation of heat shock response; interaction with nascent chain polypeptides; functions in interorganellar transport; roles in signal transduction; refolding of denatured proteins *in vitro*; roles in cell cycle and proliferation; anti-apoptotic activity; potential antigen-presenting molecule in tumour cells
	S.cervisiae	Ssa1-4,	cytosol		
		Ssb1,2	ER,		
		Kar2, Ssc1	mitochondria		
	Humans				
		Hsc70, Hsp70	cytosol,		
		BIP, mHsp70	nucleus		
			ER,		
			mitochondria		
**Hsp60**	E.coli	GroEL/ES	cytosol	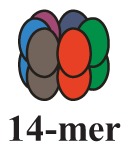	Roles in folding and stability of denatured protein *in vitro*; cofactor in diverse proteolytic systems; role in the assembly of bacteriophages and plant proteins (Rubisco)
	S.cervisiae	Hsp60	mitochondria		
	Plants	Cpn60	chloroplast		
	Humans	Hsp60	mitochondria		
**Hsp40**	E.coli	dnaJ	cytosol	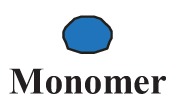	Essential co-chaperone for Hsp70 ATPase activity, substrate binding and release
	S.cervisiae	Ydj1	cytosol		
	Humans	Hdj1, Hdj2	nucleus		
**Small Hsps**	E.coli	IbpA, IpbB	cytosol	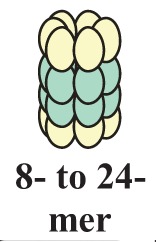	Inhibition of *in vitro* protein aggregation and heat inactivation in S. cervisiae; Role in cell thermotolerance through stabilization of actin microfilaments; anti-apoptotic activity
	Humans	Hsp27	cytosol		
		crystallin	cytosol		

## Abbreviations

AD: activation domain
AIF: apoptosis inducible factor
AMPK: AMP-activated protein kinase
Apaf-1: apoptotic protease activating factor 1
ASK-1: apoptosis signal-regulating kinase 1
BAG: Bcl2-associated athanogene
CAS: cellular apoptosis susceptibility protei
CBP: CREB binding protein
CEBPB: CCAAT/enhancer binding protein beta
CARD: caspase recruitment domain
CHIP: C-terminus of Hsp70-interacting protein
CKII: casein kinase II
CamKII: calcium/calmodulin kinase II
CTMP: carboxyl-terminal modulator protein
DBD: DNA binding domain
Diablo: IAP-binding mitochondrial protein
DSG: deoxyspergualin
ERK: extracellular regulated MAP kinase
FADD: Fas-associated death domain protein
FLIP: Flice-like inhibitor protein
GSK3β: glycogen synthase kinase 3 beta
HOP: Hsc70/Hsp90-organizing protein
HR: heptad repeat
HSE: heat shock elements
HSF: heat shock factor
HSP: heat shock protein
HSR: heat shock response
IkB: inhibitor of NF-kB
KNK437: N-formyl-3,4-methylenedioxy-benzyldiene-γ-butyroletam
JNK: Janus kinase
LAMP: lysosome-associated membrane protein
LMP: lysosomal membrane permeabilization
MAPK: mitogen activated protein kinase
MDSC: myeloid-derived suppressor cells
Mcl-1: myeloid leukaemia sequence 1
MKP: mitogen-activated protein kinase phosphatase
MRP: multidrug resistance protein
NEF: nucleotide exchanging factor
NF-kB: nuclear factor kappa-light-chain-enhancer of activated B cells
NF-IL6: nuclear factor of IL6 gene
NOA: non-oncogene addiction
PBD: peptide binding domain
PES: 2-phenylethylenesulfonamide
PHAPI: putative HLA class II associated protein
PTM: posttranslational modification
SBD: substrate binding domain
RIP1: receptor-interacting protein 1
SEK1: SAPK/ERK kinase 1
SMAC: second mitochondrial-derived activator of caspases
STAT: signal-transducer and activator of transcription protein
STRAP: stress-responsive activator of p300
p-TEFb: positive transcription elongation factor b
TLR2: Toll-like receptor 2
TNFR1: tumour necrosis factor receptor 1
TPR: tetracopeptide repeat
TRADD: TNFR1-associated death domain protein
TRAIL: TNF-related apoptosis-inducing ligand
